# Fear learning and aversive stimuli differentially change excitatory synaptic transmission in perisomatic inhibitory cells of the basal amygdala

**DOI:** 10.3389/fncel.2023.1120338

**Published:** 2023-09-05

**Authors:** Judit M. Veres, Zsuzsanna Fekete, Kinga Müller, Tibor Andrasi, Laura Rovira-Esteban, Bence Barabas, Orsolya I. Papp, Norbert Hajos

**Affiliations:** ^1^Laboratory of Network Neurophysiology, ELRN Institute of Experimental Medicine, Budapest, Hungary; ^2^János Szentágothai School of Neurosciences, Semmelweis University, Budapest, Hungary; ^3^The Linda and Jack Gill Center for Molecular Bioscience, Indiana University Bloomington, Bloomington, IN, United States; ^4^Program in Neuroscience, Department of Psychological and Brain Sciences, Indiana University Bloomington, Bloomington, IN, United States

**Keywords:** interneurons, GABA, fear conditioning, associative learning, excitation

## Abstract

Inhibitory circuits in the basal amygdala (BA) have been shown to play a crucial role in associative fear learning. How the excitatory synaptic inputs received by BA GABAergic interneurons are influenced by memory formation, a network parameter that may contribute to learning processes, is still largely unknown. Here, we investigated the features of excitatory synaptic transmission received by the three types of perisomatic inhibitory interneurons upon cue-dependent fear conditioning and aversive stimulus and tone presentations without association. Acute slices were prepared from transgenic mice: one group received tone presentation only (conditioned stimulus, CS group), the second group was challenged by mild electrical shocks unpaired with the CS (unsigned unconditioned stimulus, unsigned US group) and the third group was presented with the CS paired with the US (signed US group). We found that excitatory synaptic inputs (miniature excitatory postsynaptic currents, mEPSCs) recorded in distinct interneuron types in the BA showed plastic changes with different patterns. Parvalbumin (PV) basket cells in the unsigned US and signed US group received mEPSCs with reduced amplitude and rate in comparison to the only CS group. Coupling the US and CS in the signed US group caused a slight increase in the amplitude of the events in comparison to the unsigned US group, where the association of CS and US does not take place. Excitatory synaptic inputs onto cholecystokinin (CCK) basket cells showed a markedly different change from PV basket cells in these behavioral paradigms: only the decay time was significantly faster in the unsigned US group compared to the only CS group, whereas the amplitude of mEPSCs increased in the signed US group compared to the only CS group. Excitatory synaptic inputs received by PV axo-axonic cells showed the least difference in the three behavioral paradigm: the only significant change was that the rate of mEPSCs increased in the signed US group when compared to the only CS group. These results collectively show that associative learning and aversive stimuli unpaired with CS cause different changes in excitatory synaptic transmission in BA perisomatic interneuron types, supporting the hypothesis that they play distinct roles in the BA network operations upon pain information processing and fear memory formation.

## Introduction

Pavlovian fear conditioning is one of the most used behavioral paradigms, which serves as a model to study the basic cellular and circuit mechanisms underlying associated learning in neural networks. In this paradigm, an aversive stimulus (unconditioned stimulus, US) is presented together with a cue (conditioned stimulus, CS), resulting in a formed association between these signals wherein the animal learns that the cue (i.e., the CS) predicts the US. Previous studies have shown that the amygdala plays a central role in associative fear learning, and proposed that the association of CS and US takes place in the lateral nucleus of the amygdala (LA) (Romanski et al., 1993; Fanselow and Kim, 1994; Pape and Pare, 2010). Recent studies indicate that the link between the cue and aversive stimuli may even take place in the thalamus (Barsy et al., 2020), the association then being transmitted to the LA. The LA, after processing the information in conjunction with other inputs, passes it to the basal amygdala (BA). However, recent findings also imply that the BA has a direct role in fear memory processes (Herry et al., 2008; Amano et al., 2011; Jasnow et al., 2013).

Similar to other cortical areas, the BA networks are composed of glutamatergic excitatory principal neurons and GABAergic inhibitory interneurons (Vereczki et al., 2021). Although it is known that interneurons are necessary for appropriate and balanced cortical network functions, there is no evidence so far that excitatory synaptic inputs received by interneurons show plastic changes upon fear memory formation in the BA (Lucas et al., 2016; Polepalli et al., 2020; He et al., 2021). Interneurons in the BA show high diversity in their neurochemical content, electrophysiological properties and target distributions, which endow them with distinct roles in the network functions (Hajos, 2021). Among the many interneuron types, those which target the cell body, proximal dendrites and axon initial segment can control the activity of principal neurons the most efficiently (Miles et al., 1996). These interneurons, named perisomatic-region targeting interneurons (PTIs), are composed of three main types. Axo-axonic cells (AAC) often express parvalbumin (Vereczki et al., 2021) and target the axon initial segments of principal neurons (Somogyi, 1977; McDonald et al., 2002; Veres et al., 2014), whereas two types of basket cells contact somata and proximal dendrites: parvalbumin (PVBC) and cholecystokinin (CCKBC) containing basket cells (Freund and Katona, 2007). Based on the position of their inhibitory input onto principal neurons, PTIs can very effectively inhibit and delay cell firing (Cobb et al., 1995; Woodruff and Sah, 2007; Veres et al., 2014, 2017). Thus, PTIs can tightly control the activity of the BA networks and are potentially crucial regulators of CS-US association, however the role of distinct PTI types requires clarification. There is evidence that simultaneously manipulating all PV containing cells in the LA and BA can interfere with cue-dependent fear learning in the amygdala (Wolff et al., 2014), but how AACs and PVBCs in the BA contribute to this memory process is unknown. *In vivo* recordings suggest that the US elicits high firing activity in AACs, while PVBCs show heterogenous response: some are excited while others inhibited by aversive stimuli (Bienvenu et al., 2012; Krabbe et al., 2019). Due to the lack of appropriate tools for monitoring CCKBC activity *in vivo*, it is still unknown how their firing changes upon CS-US association. As inhibitory control of principal neuron firing by PTIs is very effective (Veres et al., 2014, 2017), any change in excitatory synaptic inputs on these GABAergic cell types can potently alter the activity of the local networks as well as the BA output.

To reveal the changes in the properties of excitatory synaptic inputs on PTIs upon fear learning, we recorded miniature excitatory postsynaptic currents (mEPSC) from identified AACs, PVBCs, and CCKBCs in acute slices prepared from three groups of mice that experienced different behavioral protocols using a tone as CS and a mild electrical shock as US. One group experienced CS only, another group received independent random CS and US (unsigned US group), and the third group was presented with CS co-terminating with US (signed US group). As fear memory formation was only observed in the signed US group, with this approach we could compare the effects of fear memory formation on the excitatory synaptic transmission received by PTIs with those obtained upon the presentation of sensory inputs (auditory and noxious stimuli).

Our results showed that the excitatory synaptic inputs on all PTI types underwent changes in response to aversive stimuli, while formation of fear association caused only minor further modifications in their properties. The different alterations in mEPSC characteristics in distinct PTI types suggest that these cells fulfill different roles during aversive information processing in BA network function and fear memory formation.

## Materials and methods

### Experimental animals

All experiments were approved by the Committee for the Scientific Ethics of Animal Research (22.1/360/3/2011) and were performed according to the guidelines of the institutional ethical code and the Hungarian Act of Animal Care and Experimentation (1998; XXVIII, section 243/1998, renewed in 40/2013) in accordance with the European Directive 86/609/CEE and modified according to the Directives 2010/63/EU. Transgenic or double-transgenic male mice (6–15 weeks old) expressing eGFP under the control of the Pvalb promoter (BAC-PV-eGFP, *n* = 19, Meyer et al., 2002), expressing DsRed under the Cck promoter (BAC-CCK-DsRed, *n* = 16, Mate et al., 2013), or expressing both eGFP and DsRed controlled by Pvalb and Cck promoters, respectively, were used (*n* = 4). Mice were housed in groups of 4–6 in the animal facility on a 12 h light/dark cycle under controlled temperature (26.5°C). Four days before the experiments, mice were kept individually to avoid cross-influence of stress levels in behavioral experiments.

### Behavioral tests

Cue-dependent fear conditioning consisted of a chamber with black dotted white background, slightly curved walls, metal rod floor, white illumination and was cleaned with 70% ethanol (context A). First, mice were allowed to habituate to this context for 5 min at Zeitgeber time (ZT) 2–3 h, then returned to their home cage. After 1 h, mice were transferred back to context A, where, after a 120 s-long acclimation period, three different protocols were used. (1) only CS group (*n* = 14): CS (7.5 kHz sound for 20 s) was presented 7 times without US (with 110 ± 23 s intervals; mean ± SD); (2) unsigned US group (*n* = 12): 7 CS and 7 US (mild electrical shocks, 2 mA for 1 s) were presented randomly [with 111 ± 21 s intervals for CS and 110 ± 33 s intervals for US (mean ± SD)]; (3) signed US group (*n* = 13): 20 s-long CS presentations were co-terminated with the 1 s-long US, pairs repeated 7 times at random intervals (110 ± 23 s; mean ± SD). On the next day at ZT 1–2 h, for testing cued fear expression, after a 120 s-long acclimation, mice were subjected to a 20 s-long CS in a novel context (context B: square chamber with white background, paper floor, red illumination, cleaned with 1% acetic acid). Freezing (as an index of fear) was *post hoc* measured manually on video recordings with an in-house software (H 77, courtesy of Prof. József Haller, Institute of Experimental Medicine, Budapest, Hungary) by trained observers blind to the animal treatment. Freezing was defined as no visible movement of the body except that required for respiration. Freezing levels are expressed as a percentage (duration of freezing within the CS/total time of the CS or duration of freezing during baseline/total time of the baseline, respectively).

### Slice preparation

Immediately after the fear expression test, mice were transferred to the anesthetizing chamber and deeply anesthetized with isoflurane and decapitated for acute slice preparation. The brain was quickly removed and placed into ice-cold solution containing (in mM): 252 sucrose, 2.5 KCl, 26 NaHCO_3_, 0.5 CaCl_2_, 5 MgCl_2_, 1.25 NaH_2_PO_4_, 10 glucose, bubbled with 95% O_2_/ 5% CO_2_ (carbogen gas). Horizontal slices of 200-μm thickness containing the BA were prepared with a Leica VT1200S vibratome and kept in an interface-type holding chamber filled with artificial cerebrospinal fluid (ACSF) at 36°C that gradually cooled down to room temperature. ACSF contained the following (in mM): 126 NaCl, 2.5 KCl, 1.25 NaH_2_PO_4_, 2 MgCl_2_, 2 CaCl_2_, 26 NaHCO_3_, and 10 glucose, bubbled with carbogen gas.

### Electrophysiological recordings

After at least 1 h incubation, slices were transferred to a submerged type recording chamber perfused with ACSF (32–34°C) containing 0.5 μM tetrodotoxin (TTX, Hello Bio) and 100 μM picrotoxin (Sigma-Aldrich) with the flow rate of approximately 2–2.5 mL/min. Recordings were performed under visual guidance using differential interference contrast microscopy (Olympus BX61W or Nikon FN-1) with a 40x objective. Neurons expressing eGFP or DsRed were visualized with the aid of a mercury arc lamp and detected with a CCD camera (Hamamatsu Photonics or Andor Zyla). Patch pipettes (4–7 MΩ) for whole-cell recordings were pulled from borosilicate capillaries with inner filament (thin walled, OD 1.5) using a DMZ-Universal Puller (Zeitz Instruments) or using a P1000 pipette puller (Sutter Instruments). The internal solution contained (in mM): 110 K-gluconate, 4 NaCl, 2 Mg-ATP, 20 HEPES, 0.1 EGTA, 0.3 GTP (sodium salt), 10 phosphocreatine, 0.2% biocytin, and 0.1 spermine adjusted to pH 7.3 using KOH, with an osmolarity of 290 mOsm/L. Neurons were recorded in whole-cell mode at a holding potential of −65 mV with 9–15 MΩ series resistance (Rs mean ± SD, in MΩ: PVBC only CS: 11.29 ± 1.30, unsigned US: 11.23 ± 1.76, signed US: 10.49 ± 0.81; CCKBC only CS: 10.85 ± 1.71, unsigned US: 10.24 ± 1.23, signed US: 10.28 ± 1.53; AAC only CS: 11.63 ± 1.80, unsigned US: 11.30 ± 1.58, signed US: 11.29 ± 1.08). Recordings were performed with a Multiclamp 700B amplifier (Molecular Devices), low-pass filtered at 3 kHz, digitized at 10–50 kHz, and not corrected for junction potential. Data acquisition software EVAN 1.3 (courtesy of Professor Istvan Mody, Department of Neurology and Physiology, University of California, Los Angeles, CA) or Clampex 10.4 (Molecular Devices) were used for recordings. mEPSC analysis was performed on the recordings obtained between 5 and 10 min after establishing whole cell configuration. During the analyzed time period (30–120 s) series resistance did not change more than 10%. During the offline analysis with Clampfit 10.4, individual miniature events were detected automatically, followed by visual inspection of each detected event and those missed by the algorithm. Only events with peak amplitude higher than 10 pA were considered as mEPSCs. Peak amplitude and inter-event interval of mEPSCs were measured in Clampfit 10.4 (n = approximately 200 consecutive events/neuron) and analyzed with Origin 2021. Statistical analysis was performed on the pooled datasets in each group. mEPSC kinetics were analyzed on the average trace of approximately 150 selected events. 10–90% of rise time was measured with Clampfit 10.4, the decay time constant (tau) was calculated by fitting an exponential curve on the average trace in Origin 2021.

### Immunostainings for identification of the recorded cells

After the recordings, slices were fixed overnight in 4% paraformaldehyde (PFA) in 0.1 M phosphate buffer (PB, pH 7.4). Biocytin-filled recorded cells were visualized with Alexa488-conjugated streptavidin (1:10.000, Molecular Probes). For CCKBC identification, immunostaining using guinea pig anti-CB1 (1,1,000; Frontier Institute, CB1-GP-Af530) or rabbit anti-CB1 (1,1,000, Cayman chemical, # 10006590), visualized either with Alexa405-coupled donkey anti-guinea pig secondary antibody (1:500, Jackson) or Alexa647-coupled donkey anti-rabbit secondary antibody (1,500, Jackson) was performed on biocytin-filled DsRed+ cells, and only those with CB1 receptor expression on their axonal boutons were included in the study. To distinguish PVBCs and AACs, immunostaining against calbindin was performed in biocytin-filled eGFP+ cells (rabbit anti-calbindin 1:3000 (Swant, CB-38a) or chicken anti-calbindin, 1:1000 (SYSY #214006), revealed with Cy3-coupled donkey-anti rabbit or chicken secondary antibodies, respectively, 1:500, Jackson). Interneurons with calbindin expression in their somata and/or axon terminals were considered BCs (Vereczki et al., 2016). AACs were defined if they showed no immunoreactivity for calbindin and displayed characteristic cartridges of terminals surrounding putative axon initial segments (AISs), that were visualized with Ankyrin G staining in case for 7 AACs (rabbit-anti Ankyrin G, 1:100, Santa Cruz sc-28,561, visualized with Cy3-coupled donkey anti-rabbit antibody, Jackson) (Gulyás et al., 2010; Veres et al., 2014). Confocal images were taken using a Nikon C2 microscope using CFI Super Plan Fluor 20X objective (N.A. 0.45; z step size: 1 μm, xy: 0.31 μm/pixel) and CFI Plan Apo VC60X Oil objective for higher magnification (N.A. 1.40; z step size: 0.13 μm, xy: 0.08 μm/pixel).

### Statistical analysis

As data showed non-normal distribution according to the Shapiro–Wilk test, Kruskal– Wallis ANOVA (K-W ANOVA) with *post hoc* Dunn’s test was used. The relationship between mEPSC properties and freezing levels was tested with Pearson’s r correlation and ANOVA. All statistics were performed using OriginPro 2018 and Origin 2021.

## Results

### Separation of the effects of fear memory formation and sensory inputs

**Figure 1:**
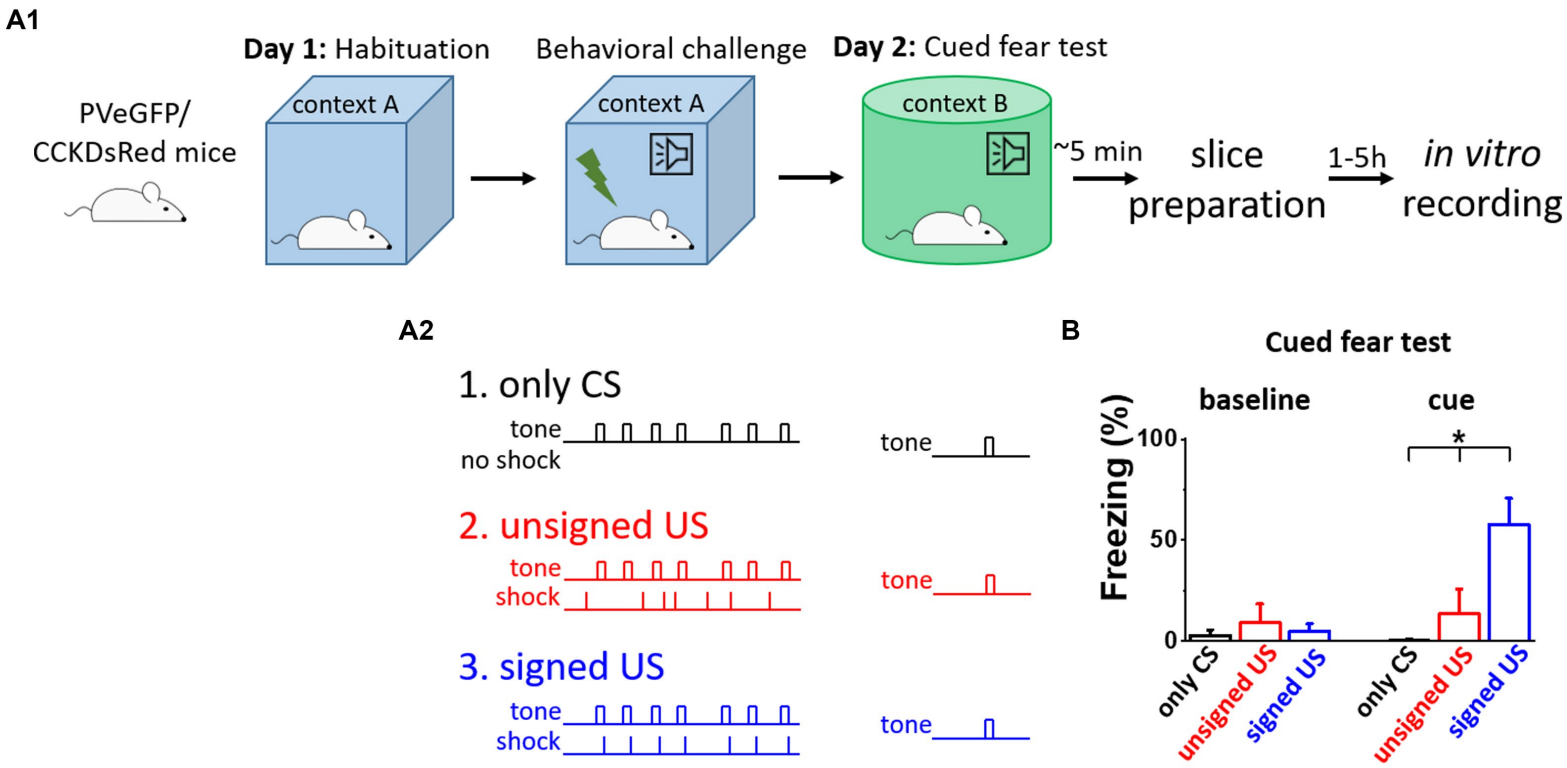
Fear memory effectively formed upon signed US presentation, but not in other conditions. **(A**_**1**_**)** Experimental design. **(A**_**2**_**)** The three mouse groups with different combinations of CS (tone) and US (shock) presentation. **(B)** Freezing tested on the consecutive day was elevated only in the signed US group, therefore the other two conditioning paradigms can serve as assessments of the effects of the sensory inputs on the network. Asterisk indicates significant difference (K-W ANOVA *p* = 3×10^−6^, only CS vs. unsigned US *p* = 0.39, only CS vs. signed US *p* = 2×10^−6^, unsigned US vs. signed US *p* = 0.003). Box represents mean, whiskers SEM. Only CS group *n* = 14, unsigned US group *n* = 12, signed US group *n* = 13.

To distinguish between the effects of fear memory formation and the CS/US presentations on the excitatory synaptic inputs of BA PTIs, three different behavioral paradigms were used (Figure 1A). First, to test the behavioral consequences of the CS presentation, mice were subjected to the CS (tones) without the US (shocks, Figure 1A_2_, only CS group, black). As expected, these mice showed no elevated freezing levels upon the CS demonstration the next day in a different context (Figure 1B, black). Then, to test the effects of CS and US without association, tones and shocks were presented randomly during conditioning (Figure 1A_2_, unsigned US group, red). In this group, the delivery of the US was not signed by the CS, therefore, the association between CS and US did not form, as demonstrated by the lack of freezing upon the cue presentation the next day (Figure 1B, red). In contrast, when tones were co-terminated with mild electrical shocks, i.e., the oncoming US was signed by a CS (Figure 1A_2_, signed US group, blue), the fear memory was formed. The results of the CS-US association were clear when the next day the CS presentation alone induced significant freezing in a different context (Figure 1B cue, K-W ANOVA *p* = 3×10^−6^), while there was no difference during the baseline period (Figure 1B baseline, K-W ANOVA *p* = 0.29). Thus, in line with previous findings, pairing a CS with a US results in lasting changes in neuronal networks, assessed at the behavioral level (Ledoux, 2003). Importantly, there was no difference in the cue evoked freezing levels of BAC-PV-eGFP and BAC-CCK-DsRed mice in the unsigned US and signed US group (Mann–Whitney test, *p* = 0.78 and *p* = 0.69, respectively), indicating that distinct BAC insertion in the two mouse lines does not compromise the fear memory porcesses. Our experimental design, therefore, allows the separation of the consequences of fear memory formation from those caused by the sensory signals via *ex vivo* investigations using the three mouse groups.

### Excitatory synaptic inputs in PVBCs are reduced upon the US presentation

**Figure 2:**
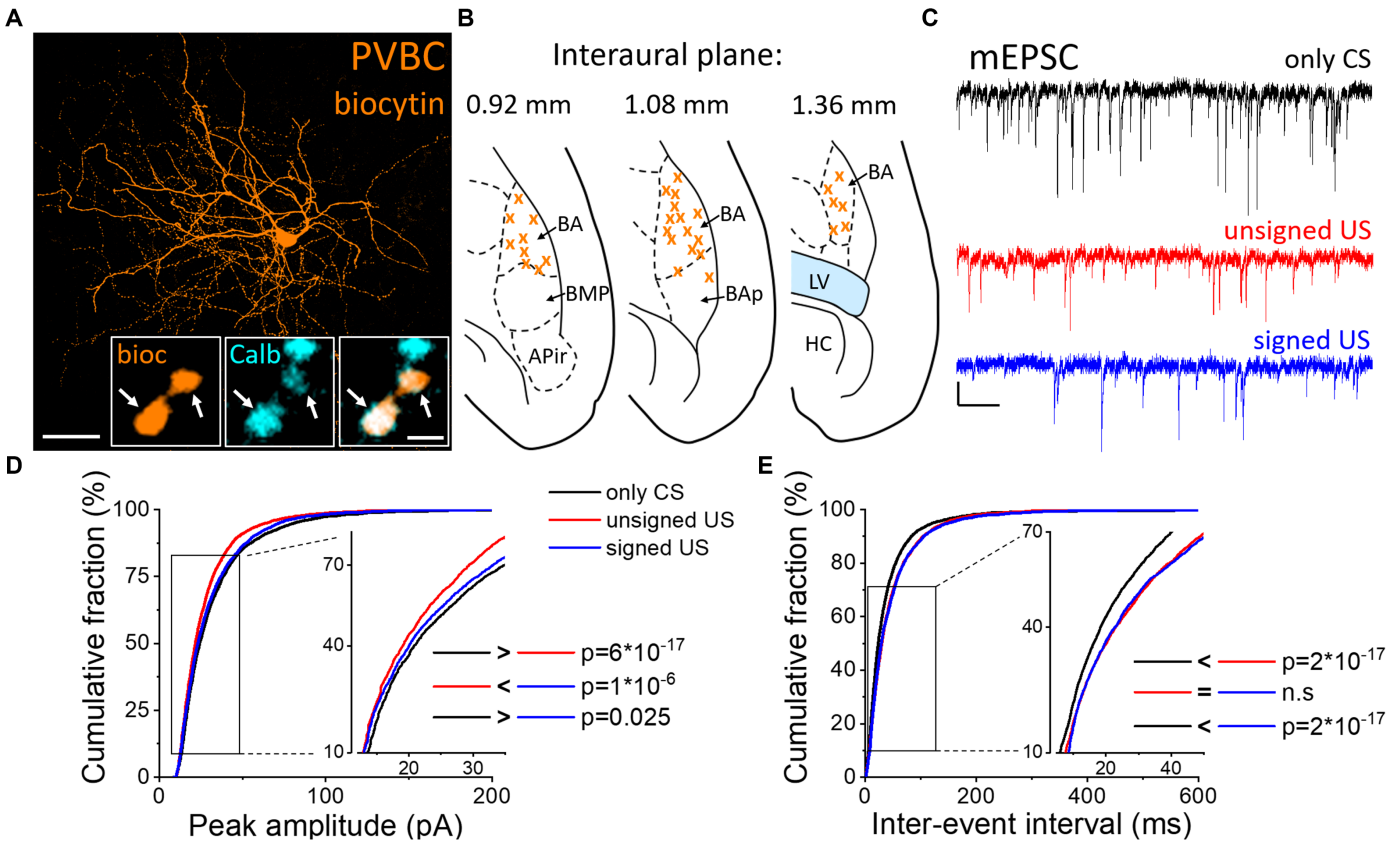
Differences in mEPSC properties in PVBCs recorded in slices prepared from the three groups of mice. **(A)** Maximum z intensity projection image of a biocytin-filled PVBC. Inset: Axon terminals of PVBCs (orange) are immunopositive for calbindin (Calb, cyan), a neurochemical marker that distinguishes PVBCs from AACs in the BA. Arrows indicate colocalization. Scales: 50 μm and 1 μm (inset). **(B)** Position (orange x) of the recorded PVBC depicted on schematic drawings representing horizontal brain sections [based on Paxinos (2012)]. Out of the 50 recorded cells, only 30 randomly selected interneurons are shown for clarity. APir: piriform amygdalar area, BA: basal amygdala, BAp: posterior part of the basal amygdala, BMP: basomedial amygdala, posterior part, HC: hippocampus, LV: lateral ventricle. **(C)** Representative traces of miniature excitatory postsynaptic current (mEPSC) recordings in the presence of 0.5 μM tetrodotoxin (TTX) and 100 μM picrotoxin in the three groups. Scales: 20 pA (y) and 100 ms (x). **(D,E)** Cumulative distribution of mEPSC peak amplitudes **(D)** and inter-event intervals **(E)**; data pooled from all cells in each group. Graphs in inserts are plotted using a normal probability Y axis. *p* values show the result of K-W ANOVA *post hoc* Dunn’s tests (see details in Table 1). n.s., non-significant difference.

To test whether excitatory synaptic inputs in different PTI types are capable of plastic changes, acute brain slices containing the amygdala were prepared immediately after cued fear testing. PV neurons in the BA were visually targeted based on their eGFP expression and their calbindin content was confirmed *post hoc* (Figures 2A, B). Calbindin is a neurochemical marker for PVBCs in the rodent amygdala that separates these interneurons from PV AACs (Bienvenu et al., 2012; Vereczki et al., 2016). mEPSCs were recorded in whole-cell patch-clamp mode in the presence of 0.5 μM tetrodotoxin (TTX, voltage-gated Na^+^ channel blocker) and 100 μM picrotoxin (GABA_A_ receptor antagonist) in slices from the three behavioral groups challenged differently (Figure 2C; Tables 1, 2). To evaluate the changes in the properties of synaptic inputs in PVBCs mEPSC parameters recorded from all cells were pooled in each group separately and analyzed (Figure 2D). The distribution of mEPSC amplitudes in PVBCs sampled in the three groups showed significant differences (Figure 2D, K-W ANOVA *p* = 1×10^−16^); we found a 11% decrease in mEPSC amplitudes in the unsigned group when compared to the only CS group (Dunn’s test *p* = 6×10^−17^) and a 5% decrease when we compared the signed US to the only CS group (Dunn’s test *p* = 0.025). Interestingly, there was a significant increase (7%) in this mEPSC feature in PVBCs if we compared those that were recorded in the unsigned and signed US groups (Dunn’s test *p* = 1×10^−6^). This observation implies that the US itself can elicit changes in mEPSC peak amplitudes in PVBCs, but the associated learning subsides those changes. When we compared the inter-event intervals (IEI) of mEPSCs (Figure 2E) we found significant changes among groups (K-W ANOVA *p* = 3×10^−22^). When compared to the only CS group, the unsigned US presentation led to a 26% (Dunn’s test *p* = 2×10^−17^) increase in IEIs (i.e., reduced the rate), and the signed US group also showed a 23% increase (Dunn’s test *p* = 2×10^−17^). There was no difference in mEPSC rates in the unsigned and signed US group (Dunn’s test *p* = 0.3), implying that the effect of CS and US association on this mEPSC characteristic is indistinguishable from those that are caused by the independent presentation of CS and US. Rise time and decay kinetics of mEPSCs were not different in the three paradigms (Table 2, K-W ANOVA *p* = 0.758 and *p* = 0.598, respectively). Taken together, these results suggest that the US presentation decreases mEPSC amplitudes and their occurrence in PVBCs, but fear memory formation may cause a slighter reduction in the amplitude of their excitatory synaptic inputs in comparison to CS presentation only.

**Table 1:** Data and statistical analysis of mEPSC amplitude and inter-event interval in the recorded PTIs.

Cell type	Variable	Group	Cell number (mouse number)	Value (median ± IQ range)	Group comparison *p* value (K-W ANOVA)	Paired comparison	*p* value (Dunn’s test)	Change
PVBC	Peak amplitude (pA) (Figure 2D)	Only CS	17 (7)	24.35 ± 21.41	1*10^−16^	Only CS vs. unsigned US	6*10^−17^	11% ↓
		Unsigned US	17 (6)	21.66 ± 16.55		Only CS vs. signed US	0.0251	5% ↓
		Signed US	16 (7)	23.11 ± 20.85		Unsigned US vs. signed US	1*10^−6^	7% ↑
	IEI (ms) (Figure 2E)	Only CS	17 (7)	23.70 ± 34.90	3*10^−22^	Only CS vs. unsigned US	2*10^−17^	26% ↑
		Unsigned US	17 (6)	30.00 ± 43.60		Only CS vs. signed US	2*10^−17^	23% ↑
		Signed US	16 (7)	29.24 ± 44.55		Unsigned US vs. signed US	1	–
CCKBC	Peak amplitude (pA) (Figure 3D)	Only CS	23 (6)	17.13 ± 7.12	0.001	Only CS vs. unsigned US	0.124	–
		Unsigned US	24 (6)	17.32 ± 7.67		Only CS vs. signed US	9*10^−4^	3% ↑
		Signed US	21 (6)	17.58 ± 7.96		Unsigned US vs. signed US	0.303	–
	IEI (ms) (Figure 3E)	Only CS	23 (6)	83.31 ± 137.0	0.175			
		Unsigned US	24 (6)	85.35 ± 144.75				
		Signed US	21 (6)	83.75 ± 131.30				
AAC	Peak amplitude (pA) (Figure 4D)	Only CS	20 (7)	18.06 ± 10.51	0.406			
		Unsigned US	14 (5)	17.76 ± 10.64				
		Signed US	21 (7)	18.19 ± 11.17				
	IEI (ms) (Figure 4E)	Only CS	20 (7)	58.75 ± 104.00	0.031	Only CS vs. unsigned US	1	–
		Unsigned US	14 (5)	56.45 ± 96.73		Only CS vs. signed US	0.026	7% ↓
		Signed US	21 (7)	54.45 ± 90.50		Unsigned US vs. signed US	0.465	−

**Table 2:** Data and statistical analysis of mEPSC kinetic features recorded in PTIs.

Cell type	Variable	Group	Cell number (mouse number)	Value (median ± IQ range)	Group comparison p value (K-W ANOVA)	Paired comparison	*p* value (Dunn’s test)	Change
PVBC	Rise time 10–90% (ms)	Only CS	17 (7)	0.28 ± 0.07	0.758	Only CS vs. unsigned US		
		Unsigned US	17 (6)	0.31 ± 0.06		Only CS vs. signed US		
		Signed US	16 (7)	0.34 ± 0.09		Unsigned US vs. signed US		
	Decay time constant (ms)	Only CS	17 (7)	0.94 ± 0.38	0.598	Only CS vs. unsigned US		
		Unsigned US	17 (6)	1.01 ± 0.40		Only CS vs. signed US		
		Signed US	16 (7)	1.06 ± 0.4		Unsigned US vs. signed US		
CCKBC	Rise time 10–90% (ms)	Only CS	23 (6)	0.60 ± 0.20	0.895	Only CS vs. unsigned US		
		Unsigned US	24 (6)	0.61 ± 0.09		Only CS vs. signed US		
		Signed US	21 (6)	0.60 ± 0.11		Unsigned US vs. signed US		
	Decay time constant (ms)	Only CS	23 (6)	1.91 ± 0.58	0.006	Only CS vs. unsigned US	0.004	17% ↓
		Unsigned US	24 (6)	1.57 ± 0.41		Only CS vs. signed US	0.375	–
		Signed US	21 (6)	1.80 ± 0.46		Unsigned US vs. signed US	0.352	–
AAC	Rise time 10–90% (ms)	Only CS	20 (7)	0.26 ± 0.09	0.230	Only CS vs. unsigned US		
		Unsigned US	14 (5)	0.33 ± 0.08		Only CS vs. signed US		
		Signed US	21 (7)	0.32 ± 0.09		Unsigned US vs. signed US		
	Decay time constant (ms)	Only CS	20 (7)	0.84 ± 0.20	0.289	Only CS vs. unsigned US		
		Unsigned US	14 (5)	0.93 ± 0.18		Only CS vs. signed US		
		Signed US	21 (7)	0.91 ± 0.24		Unsigned US vs. signed US		

### Increased amplitude and decreased decay time constant of mEPSCs in CCKBCs upon US presentation

**Figure 3:**
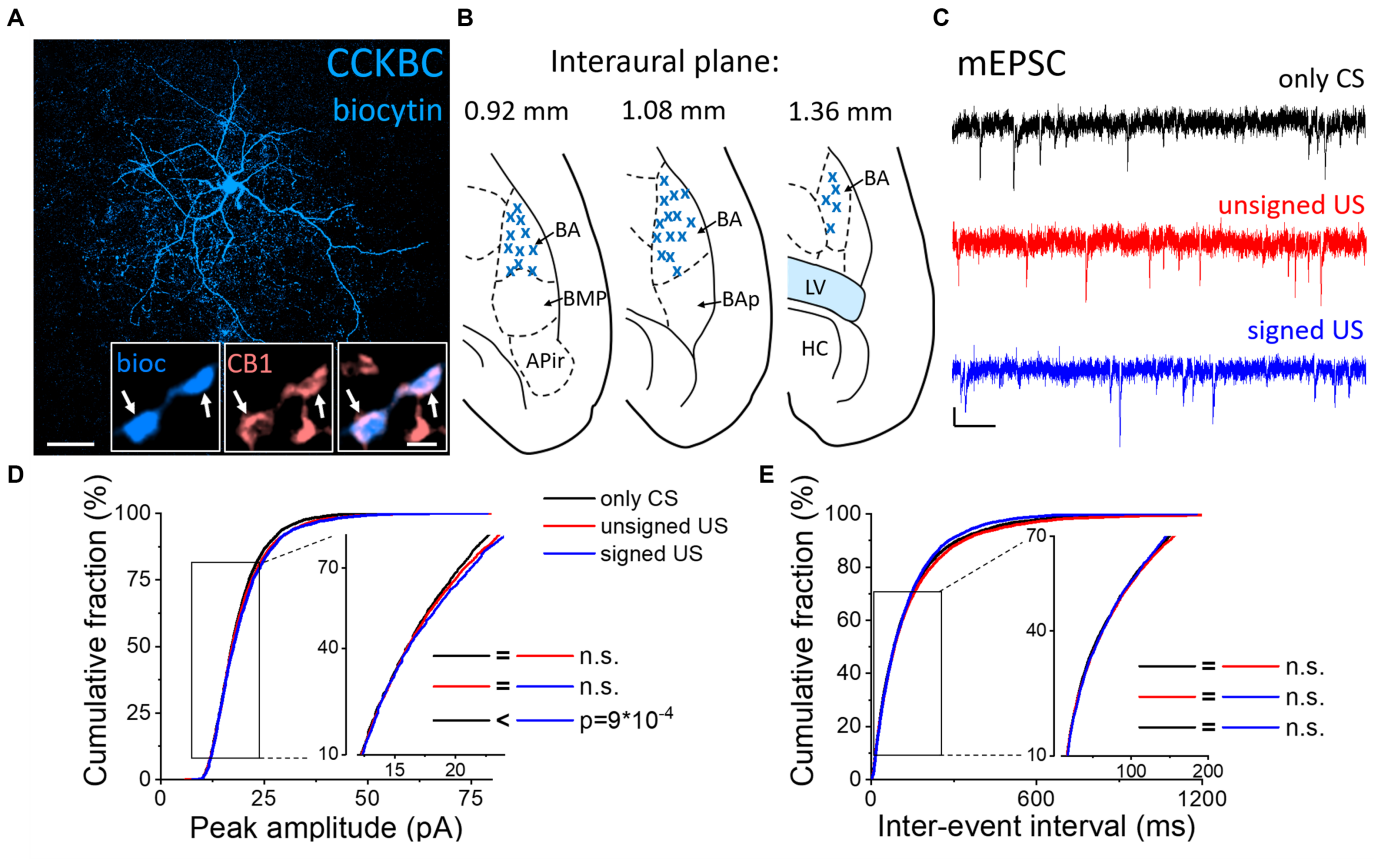
Increased amplitude of mEPSCs in CCKBCs upon US presentation **(A)** Maximum z intensity projection image of a biocytin-filled CCKBC. Inset: Axon terminals of same CCKBC (blue) are immunopositive for type one cannabinoid receptors (CB1, pink). Arrows indicate colocalization. Scales: 50 μm and 1 μm (inset). **(B)** Position (blue x) of the recorded CCKBC depicted on schematic drawings representing horizontal brain sections [based on Paxinos (2012)]. Out of the 68 recorded cells, only 30 randomly selected interneurons are shown for clarity. APir: piriform amygdalar area, BA: basal amygdala, BAp: posterior part of the basal amygdala, BMP: basomedial amygdala, posterior part, HC: hippocampus, LV: lateral ventricle. **(C)** Representative traces of miniature excitatory postsynaptic current (mEPSC) recordings in the presence of 0.5 μM TTX and 100 μM picrotoxin from the three groups. Scales: 10 pA (y) and 100 ms (x). **(D,E)** Cumulative distribution of mEPSC peak amplitudes **(D)** and inter-event intervals **(E)**; data pooled from all cells in each group. Graphs in inserts are plotted using a normal probability Y axis. *p* values show the result of K-W ANOVA *post hoc* Dunn’s tests (see details in Table 1). n.s., non-significant difference.

Next, we assessed whether the excitatory synaptic inputs in the other main basket cell type, CCKBCs, are also capable of plastic changes in our paradigms. To selectively record from these cells, a CCK-DsRed mouse strain was used (Mate et al., 2013; Rovira-Esteban et al., 2017) to visually target CCKBCs based on their DsRed content (Figures 3A, B; Vereczki et al., 2016). After recordings, CB1 content of axon terminals was confirmed with immunolabeling (Figure 3A insets). mEPSCs were recorded (Figure 3C; Tables 1, 2) and analyzed by the same methods as in PVBCs described above. The evaluation of changes in mEPSC characteristics recorded in CCKBCs showed a difference in their peak amplitudes (Figure 3D, K-W ANOVA *p* = 0.001). There was a slight (3%) but significant increase in mEPSC peak amplitudes in the signed US groups when compared to the only CS controls (Dunn’s test *p* = 2×10^−17^). However, there was no significant difference between the only CS vs. unsigned US (Dunn’s test *p* = 0.12) and unsigned US vs. signed US comparisons (Dunn’s test *p* = 0.30). Interestingly, we could not find any difference in the IEI of mEPSCs (Figure 3E, K-W ANOVA *p* = 0.175). Regarding the kinetic properties of mEPSCs (Table 2), the rise time was not different in the three paradigms (K-W ANOVA *p* = 0.895), however, there was a significant 17% decrease in the decay time constant when we compared the only CS group to the unsigned US group (Dunn’s test *p* = 0.004). Taken together, these results show that the fear learning increases the amplitude of mEPSC and the unsigned US accelerates the mEPSC decaying phase in the CCKBC population.

### Excitatory synaptic inputs in AACs change only upon fear memory formation

**Figure 4:**
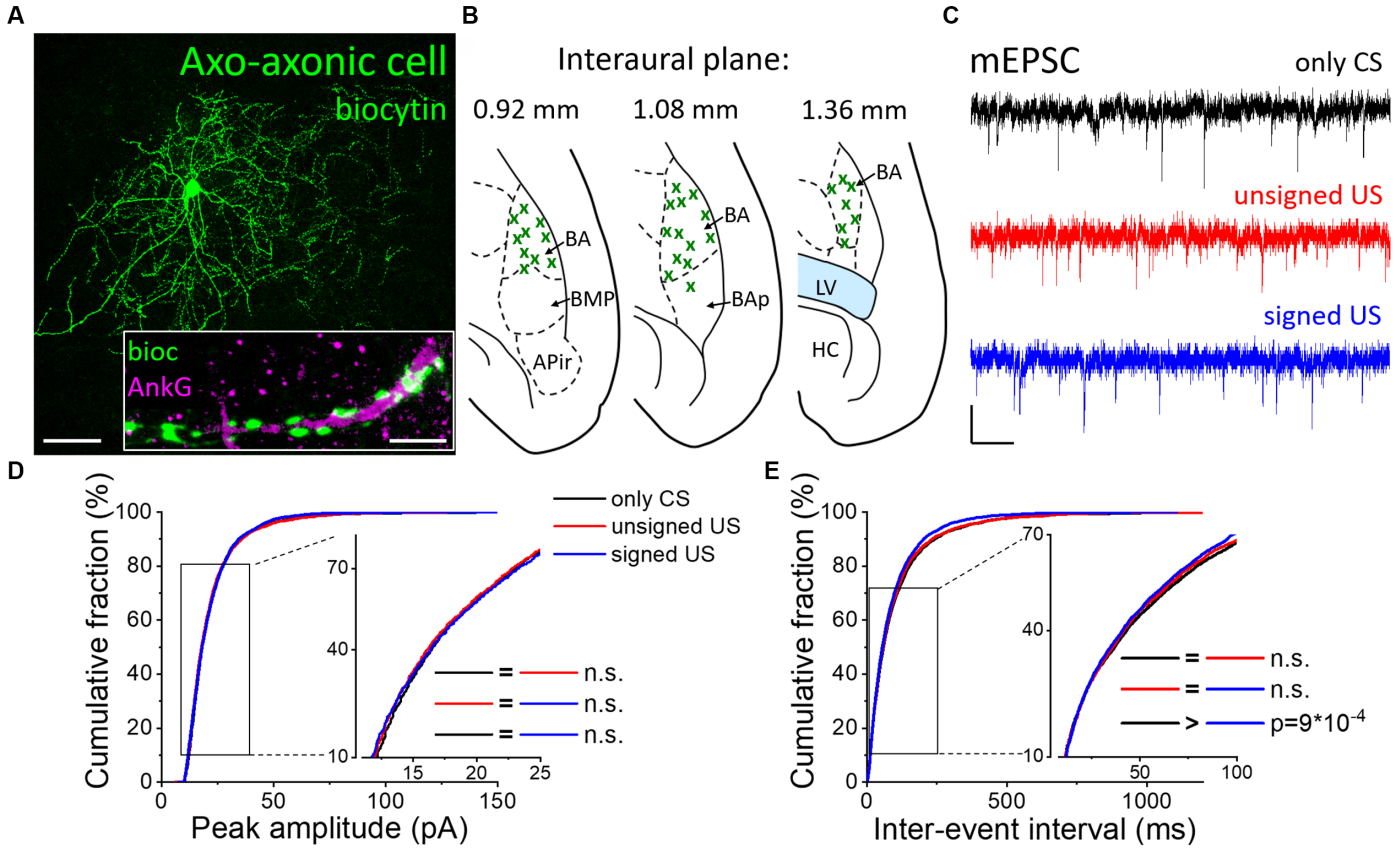
Excitatory synaptic inputs in AACs change only upon the signed US presentation. **(A)** Maximum z intensity projection image of a biocytin-filled AAC. Inset: Axon terminals of the same AAC (green) form a characteristic cartridge along an Ankyrin-G (magenta) labeled axon initial segment. Scales: 50 μm and 5 μm (inset). **(B)** Position (green x) of each recorded AAC depicted on schematic drawings representing horizontal brain sections [based on Paxinos (2012)]. Out of the 55 recorded cells, only 30 randomly selected interneurons are shown for clarity. APir: piriform amygdalar area, BA: basal amygdala, BAp: posterior part of the basal amygdala, BMP: basomedial amygdala, posterior part, HC: hippocampus, LV: lateral ventricle. **(C)** Representative traces of miniature excitatory postsynaptic current (mEPSC) recordings in the presence of 0.5 μM TTX and 100 μM picrotoxin obtained in AACs sampled from the three groups. Scales: 10 pA (y) and 100 ms (x). **(D,E)** Cumulative distribution of mEPSC peak amplitudes **(D)** and inter-event intervals **(E)**; data pooled from all cells in each group. Graphs in inserts are plotted using a normal probability Y axis. *p* values show the result of K-W ANOVA *post hoc* Dunn’s tests (see details in Table 1). n.s., non-significant difference.

Besides the two basket cell types, AACs are the third PTI type that are capable to efficiently control the spiking activity of principal neurons (Veres et al., 2014). Therefore, any change in the excitatory synaptic inputs of AACs as a consequence of fear conditioning could be pivotal in the accomplishment of their functions. AACs were targeted in the BA based on their eGFP content in PV-eGFP animals and were separated *post hoc* from PVBCs based on their characteristic axonal cartridges formed around axon initial segments that can be visualized by Ankyrin-G staining (Figures 4A, B) and the absence of calbindin immunolabeling in their somata and axon terminals. mEPSCs were recorded (Figure 4C; Tables 1, 2) and analyzed as described above. Unlike basket cells, excitatory synaptic inputs in AACs did not show change in their amplitude (Figure 4D, K-W ANOVA *p* = 0.406), however, there was a slight but significant change in the rate of mEPSCs ((Figure 4E, K-W ANOVA *p* = 0.031): in the signed US group the rate of mEPSCs were 7% higher than in the only CS group (Dunn’s test *p* = 0.026). Rise time and decay kinetics of mEPSCs were not different in the three paradigms (Table 2, K-W ANOVA *p* = 0.23 and *p* = 0.289, respectively). Taken together, our data indicate that there is a unique increase in mEPSC rates in AACs upon signed US presentation that was not present in any other PTI type.

### Fear learning strength does not correlate with mEPSC properties

**Figure 5:**
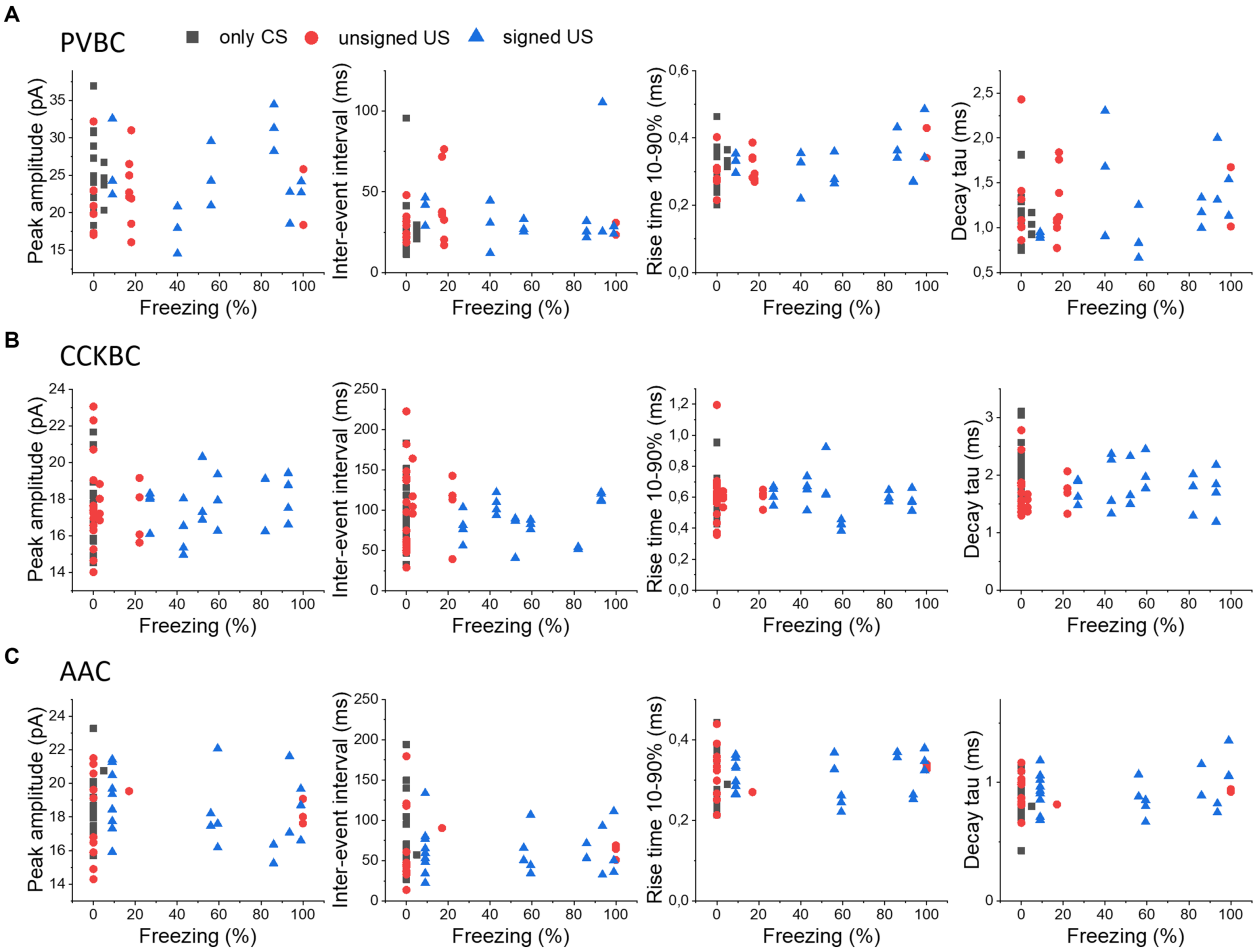
No correlation in the properties of mEPSCs in PTIs and the expression of fear levels. The median values of peak amplitude, inter-event interval, rise time 10–90% and decay time constant (tau) of mEPSCs for each cell are shown as a function of the freezing levels upon cue presentation in PVBCs **(A)**, CCKBCs **(B)**, and AACs **(C)**.

As the strength of fear memory learning can be variable among individual mice, assessed by the time spent in freezing, we asked the question whether the variability in freezing is reflected in the excitatory inputs of PTIs in the BA. Therefore, we compared the electrophysiological properties of mEPSCs in PTIs recorded from mice with different freezing levels upon cue presentation on the test day, as a proxy for the strength of fear learning (Figure 5). We correlated the peak amplitude, IEI, rise time and decay time constant of mEPSCs in PVBCs (Figure 5A), CCKBCs (Figure 5B) and AACs (Figure 5C), in each behavioral paradigm (only CS, unsigned US and signed US groups). We found that none of the groups showed a significant linear correlation with the freezing level of the animal (*p* > 0.05), suggesting that the level of behavioral output induced by fear learning in the individuals is not reflected in the properties of excitatory inputs at the single cell level.

## Discussion

**Figure 6:**
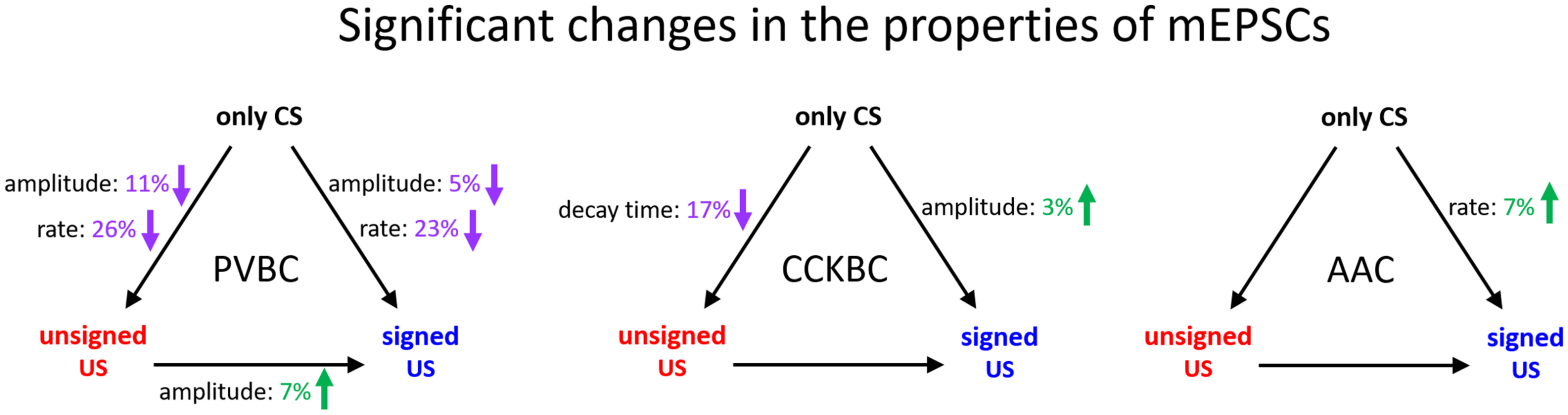
Summary of significant changes in the properties of excitatory synaptic inputs of PTIs in the BA in the three behavioral paradigm. Black arrows indicate the compared pairs, only the significant changes in mEPSC amplitude, rate and decay time are shown and are expressed in % values. Green numbers and upward arrows indicate increase, purple numbers and downward arrows indicate decrease. For the exact tests and significance values see Tables 1, 2.

In this study, we revealed cell type-specific changes in the properties of excitatory synaptic transmission in PTIs upon unsigned aversive stimulus presentation and fear memory formation in comparison to tone presentation only in the BA (Figure 6). Both the peak amplitude and rate of mEPSCs in PVBCs decreased in the unsigned and signed US paradigm when compared to the only CS group, though a smaller decrease could be observed in the amplitude of mEPSCs when the unsigned US and CS only groups were compared. AACs received excitatory synaptic inputs with shorter inter-event intervals (i.e., the mEPSC rate increased) when the US was paired to the CS compared to the only CS group. These results suggest that although these two PTI types show similarities in their PV expression and features in the membrane voltage responses (Barsy et al., 2017), their excitatory synaptic inputs are modified distinctly by fear learning, implying the different roles of PVBCs and AACs in network function. In contrast to PVBCs and AACs, CCKBCs were the only cell type showing any change in mEPSC kinetics (in decay time constant) after unpaired US protocol, with no further modification after fear memory formation. Also, in sharp contrast to PVBCs, there was a slight but significant increase in mEPSC amplitudes in the fear association paradigm when we compared it to the only CS group. Such opposite change in the synaptic transmission observed between CCKBCs and PVBCs is exemplified in multiple instances. For example, nitric oxide originating from the postsynaptic pyramidal neurons reduces the inhibitory transmission from the axon terminals of CCKBCs (Makara et al., 2007), but increases transmission at the output synapses of PVBCs (Lourenco et al., 2014). Thus, opposite alterations in CCKBC and PVBC characteristics under various circumstances may be a general rule in cortical circuits.

It has been shown that activity of interneurons changes in response to both the CS and US (Krabbe et al., 2019), therefore, in order to dissect their role in processing aversive stimuli versus fear memory formation, it is important to use a control group that was exposed to the auditory stimulus (only CS group), instead of using naïve mice as controls. To separate the effects of aversive stimuli and fear memory formation, we used the unsigned US group as an additional reference where the noxious stimulus could not be predicted by the presentation of the CS. With the use of these two groups, we demonstrated that excitatory synaptic inputs of all PTI types in the BA were modified in response to the aversive stimuli. Notably, CCKBCs were the only PTI type that showed a change in the kinetic properties of their excitatory synaptic inputs in the unsigned group, a finding that may imply a unique alteration in the excitatory input of CCKBCs after exposure to aversive stimuli. The faster decay of mEPSCs may be related to the changes in subunit composition of ionotropic glutamate receptors mediating the synaptic communication between the excitatory cells and CCKBCs. Such changes in kinetics upon environmental challenges have been described recently (Shultz et al., 2022). Our findings that mEPSC amplitudes are increased in the signed US group when compared to the only CS group may suggest that increasing excitation on CCKBCs can lead to a more potent recruitment of these interneurons upon learning. This elevated recruitment may cause an increased synaptic inhibition in the principal neuron populations that can be overcome only by those highly active, presumably coding, principal neurons that are able to trigger depolarization induced suppression of inhibition (DSI) at their GABAergic inputs from CCKBCs (Pitler and Alger, 1992; Wilson and Nicoll, 2001; Zhu and Lovinger, 2005; Losonczy et al., 2010), while the synaptic inhibition on the non-coding cells remains intact. This process, therefore, may be an efficient mechanism to improve the signal-to-noise ratio in BA networks during aversive stimulus processing (Grewe et al., 2017). Alternatively, the change in the excitatory synaptic transmission received by CCKBCs could reflect their crucial role in stress responses evoked by the unpredictable noxious stimuli, a paradigm that serves also as a stress model (Matuszewich et al., 2007). Notably, it has been previously shown that PVBCs and CCKBCs form independent, parallel inhibitory networks in the BA (Andrasi et al., 2017), a circuit motif that also supports their divergent functions in amygdala operations.

Although the extent of the changes in mEPSC properties in BA PTIs may seem a rather small, it has to be kept in mind that both PV and CCK interneurons receive thousands of glutamatergic inputs (Gulyas et al., 1999; Matyas et al., 2004) of which only a small portion is expected to be altered upon associative learning or noxious stimulus processing. This assumption is based on the observations that PTIs receive excitatory inputs from their neighbor principal neurons (Andrasi et al., 2017), a portion of which is engaged in memory processes or pain processing does not exceed typically 10–15% (Trouche et al., 2013; Senn et al., 2014; Grewe et al., 2017; Corder et al., 2019). In addition, interneurons are innervated also by extra-amygdalar excitatory afferents, e.g., from the thalamus (Krabbe et al., 2019). Although their ratio is unknown within the glutamatergic synapses received by distinct GABAergic cell types in the BA at present, these inputs may also undergo plastic changes upon fear learning (Barsy et al., 2020), thus, they can also contribute to the observed changes in this study. Moreover, not all GABAergic interneurons within a population have been found to participate in these neural processes (Bienvenu et al., 2012; Wolff et al., 2014; Krabbe et al., 2019). Thus, in a randomly sampled interneuron pool, the contribution of the excitatory inputs that underwent plastic changes in our circumstances to the entire population may be rather limited.

A previous study compared the changes of synaptic inputs on PV interneurons in the LA and BA after fear learning and found that the rate of mEPSCs in PV interneurons is changed only in the LA but not in the BA (Lucas et al., 2016). However, in that study PVBCs and AACs were not distinguished. As the excitatory synaptic inputs of these GABAergic cell types underwent different alterations after learning in the BA (Figure 6), pooling of their data may mask the changes observed between the mEPSC properties recorded in PVBCs and PV AACs. What could be the mechanisms underlying the observed decrease in mEPSC amplitudes in PVBCs upon the US presentation? Our previous study showed that excitatory synaptic inputs on PVBCs and AACs can undergo LTD in a CB1 receptor-dependent manner in the hippocampus (Peterfi et al., 2012) a mechanism that might explain the US-induced decrease in the amplitude of excitatory synaptic inputs observed in PVBCs. However, the increased mEPSC rate in AACs in the signed US group in comparison to the only CS group indicates that synaptic mechanisms underlying the changes in mEPSC properties observed in PVBCs and AACs during the different challenges are necessarily distinct.

The basolateral amygdala is thought to play a central role in fear memory processes, and PTIs in this region are crucial in regulating BA networks due to their powerful capacity to control principal neuron functions (Veres et al., 2014, 2017). Previous studies have highlighted cell type specific roles of interneurons by reporting specific responses to aversive stimuli and firing during oscillations (Bienvenu et al., 2012; Manko et al., 2012; Krabbe et al., 2019), unique sensitivity to neuromodulators (Bocchio et al., 2016; Fu et al., 2022; Mineur et al., 2022), and behavior-induced, target specific output changes (Trouche et al., 2013). Our study extends this knowledge to the level of their excitatory input by showing PTI type specific differences upon aversive stimulus processing and fear memory formation, which will help us to understand how different types of interneurons in the BA network function in health and disease.

## Data availability statement

The raw data supporting the conclusions of this article will be made available by the authors, without undue reservation.

## Ethics statement

The animal study was approved by the Animal Committee of the Institute of Experimental Medicine, Budapest, Hungary. The study was conducted in accordance with the local legislation and institutional requirements.

## Author contributions

JV: acquisition of data, analysis and interpretation of data, drafting the article. ZF, KM, TA, and LR-E: acquisition of data. OP and BB: acquisition of data, analysis and interpretation of data. NH: conception and design, analysis and interpretation of data, drafting the article. All authors contributed to the article and approved the submitted version.

## Funding

This work was supported by National Research, Development and Innovation Office (K131893 and RRF-2.3.1-21-2022-00011) and Hungarian Brain Research Program (2017-1.2.1-NKP-2017-00002).

## Conflict of interest

The authors declare that the research was conducted in the absence of any commercial or financial relationships that could be construed as a potential conflict of interest.

## Publisher’s note

All claims expressed in this article are solely those of the authors and do not necessarily represent those of their affiliated organizations, or those of the publisher, the editors and the reviewers. Any product that may be evaluated in this article, or claim that may be made by its manufacturer, is not guaranteed or endorsed by the publisher.

## Figures and Tables

**Table:** 

AAC	axo-axonic cell
ACSF	artificial cerebrospinal fluid
AnkG	Ankyrin G
APir	piriform amygdalar area
BA	basal amygdala
BAp	posterior part of the basal amygdala
BC	basket cell
BMP	basomedial amygdala, posterior part
Calb	calbindin
CB1	type 1 cannabinoid receptor
CCK	cholecystokinin
CS	conditioned stimulus
DSI	depolarization induced suppression of inhibition
eGFP	enhanced green fluorescent protein
GABA	gamma-amino butyric acid
HC	hippocampus
IEI	inter-event interval
LA	lateral amygdala
LTD	long-term depression
LTP	long-term potentiation
LV	lateral ventricle
mEPSC	miniature excitatory postsynaptic potential
PB	phosphate buffer
PFA	paraformaldehyde
PTI	perisomatic region targeting interneuron
PV	parvalbumin
TTX	tetrodotoxin
US	unconditioned stimulus
ZT	Zeitgeber time

## References

[ref1] Amano T. Duvarci S. Popa D. Pare D. (2011). The fear circuit revisited: contributions of the basal amygdala nuclei to conditioned fear. J. Neurosci. 31, 15481–15489. doi: 10.1523/JNEUROSCI.3410-11.2011 22031894 PMC3221940

[ref2] Andrasi T. Veres J. M. Rovira-Esteban L. Kozma R. Vikor A. Gregori E.. (2017). Differential excitatory control of 2 parallel basket cell networks in amygdala microcircuits. PLoS Biol. 15:e2001421. doi: 10.1371/journal.pbio.2001421 28542195 PMC5443504

[ref3] Barsy B. Kocsis K. Magyar A. Babiczky A. Szabo M. Veres J. M.. (2020). Associative and plastic thalamic signaling to the lateral amygdala controls fear behavior. Nat. Neurosci. 23, 625–637. doi: 10.1038/s41593-020-0620-z 32284608

[ref4] Barsy B. Szabo G. G. Andrasi T. Vikor A. Hajos N. (2017). Different output properties of perisomatic region-targeting interneurons in the basal amygdala. Eur. J. Neurosci. 45, 548–558. doi: 10.1111/ejn.13498, PMID: 27977063

[ref5] Bienvenu T. C. Busti D. Magill P. J. Ferraguti F. Capogna M. (2012). Cell-type-specific recruitment of amygdala interneurons to hippocampal theta rhythm and noxious stimuli in vivo. Neuron 74, 1059–1074. doi: 10.1016/j.neuron.2012.04.022, PMID: 22726836 PMC3391683

[ref6] Bocchio M. Mchugh S. B. Bannerman D. M. Sharp T. Capogna M. (2016). Serotonin, amygdala and fear: assembling the puzzle. Front. Neural Circuits 10:24. doi: 10.3389/fncir.2016.00024 27092057 PMC4820447

[ref7] Cobb S. R. Buhl E. H. Halasy K. Paulsen O. Somogyi P. (1995). Synchronization of neuronal activity in hippocampus by individual GABAergic interneurons. Nature 378, 75–78. doi: 10.1038/378075a0, PMID: 7477292

[ref8] Corder G. Ahanonu B. Grewe B. F. Wang D. Schnitzer M. J. Scherrer G. (2019). An amygdalar neural ensemble that encodes the unpleasantness of pain. Science 363, 276–281. doi: 10.1126/science.aap8586, PMID: 30655440 PMC6450685

[ref9] Fanselow M. S. Kim J. J. (1994). Acquisition of contextual Pavlovian fear conditioning is blocked by application of an NMDA receptor antagonist D,L-2-amino-5-phosphonovaleric acid to the basolateral amygdala. Behav. Neurosci. 108, 210–212. doi: 10.1037/0735-7044.108.1.210, PMID: 7910746

[ref10] Freund T. F. Katona I. (2007). Perisomatic inhibition. Neuron 56, 33–42. doi: 10.1016/j.neuron.2007.09.012 17920013

[ref11] Fu X. Teboul E. Weiss G. L. Antonoudiou P. Borkar C. D. Fadok J. P.. (2022). Gq neuromodulation of BLA parvalbumin interneurons induces burst firing and mediates fear-associated network and behavioral state transition in mice. Nat. Commun. 13:1290. doi: 10.1038/s41467-022-28928-y, PMID: 35277502 PMC8917207

[ref12] Grewe B. F. Grundemann J. Kitch L. J. Lecoq J. A. Parker J. G. Marshall J. D.. (2017). Neural ensemble dynamics underlying a long-term associative memory. Nature 543, 670–675. doi: 10.1038/nature21682, PMID: 28329757 PMC5378308

[ref13] Gulyas A. I. Megias M. Emri Z. Freund T. F. (1999). Total number and ratio of excitatory and inhibitory synapses converging onto single interneurons of different types in the CA1 area of the rat hippocampus. J. Neurosci. Off. J. Soc. Neurosci. 19, 10082–10097. doi: 10.1523/JNEUROSCI.19-22-10082.1999, PMID: 10559416 PMC6782984

[ref14] Gulyás A. I. Szabó G. G. Ulbert I. Holderith N. Monyer H. Erdélyi F.. (2010). Parvalbumin-containing fast-spiking basket cells generate the field potential oscillations induced by cholinergic receptor activation in the hippocampus. J. Neurosci. 30, 15134–15145. doi: 10.1523/JNEUROSCI.4104-10.2010, PMID: 21068319 PMC3044880

[ref15] Hajos N. (2021). Interneuron types and their circuits in the basolateral amygdala. Front. Neural Circuits 15:687257. doi: 10.3389/fncir.2021.687257, PMID: 34177472 PMC8222668

[ref16] He X. Li J. Zhou G. Yang J. Mckenzie S. Li Y.. (2021). Gating of hippocampal rhythms and memory by synaptic plasticity in inhibitory interneurons. Neuron 109:1013. doi: 10.1016/j.neuron.2021.01.014 33548174 PMC9239733

[ref17] Herry C. Ciocchi S. Senn V. Demmou L. Muller C. Luthi A. (2008). Switching on and off fear by distinct neuronal circuits. Nature 454, 600–606. doi: 10.1038/nature07166, PMID: 18615015

[ref18] Jasnow A. M. Ehrlich D. E. Choi D. C. Dabrowska J. Bowers M. E. Mccullough K. M.. (2013). Thy1-expressing neurons in the basolateral amygdala may mediate fear inhibition. J. Neurosci. 33, 10396–10404. doi: 10.1523/JNEUROSCI.5539-12.2013, PMID: 23785152 PMC3685835

[ref19] Krabbe S. Paradiso E. D'aquin S. Bitterman Y. Courtin J. Xu C.. (2019). Adaptive disinhibitory gating by VIP interneurons permits associative learning. Nat. Neurosci. 22, 1834–1843. doi: 10.1038/s41593-019-0508-y, PMID: 31636447

[ref20] Ledoux J. (2003). The emotional brain, fear, and the amygdala. Cell. Mol. Neurobiol. 23, 727–738. doi: 10.1023/A:1025048802629 14514027 PMC11530156

[ref21] Losonczy A. Zemelman B. V. Vaziri A. Magee J. C. (2010). Network mechanisms of theta related neuronal activity in hippocampal CA1 pyramidal neurons. Nat. Neurosci. 13, 967–972. doi: 10.1038/nn.2597, PMID: 20639875 PMC2921679

[ref22] Lourenco J. Pacioni S. Rebola N. Van Woerden G. M. Marinelli S. Digregorio D.. (2014). Non-associative potentiation of perisomatic inhibition alters the temporal coding of neocortical layer 5 pyramidal neurons. PLoS Biol. 12:e1001903. doi: 10.1371/journal.pbio.1001903, PMID: 25003184 PMC4086817

[ref23] Lucas E. K. Jegarl A. M. Morishita H. Clem R. L. (2016). Multimodal and site-specific plasticity of amygdala parvalbumin interneurons after fear learning. Neuron 91, 629–643. doi: 10.1016/j.neuron.2016.06.032, PMID: 27427462 PMC4975985

[ref24] Makara J. K. Katona I. Nyiri G. Nemeth B. Ledent C. Watanabe M.. (2007). Involvement of nitric oxide in depolarization-induced suppression of inhibition in hippocampal pyramidal cells during activation of cholinergic receptors. J. Neurosci. 27, 10211–10222. doi: 10.1523/JNEUROSCI.2104-07.2007, PMID: 17881527 PMC6672656

[ref25] Manko M. Bienvenu T. C. Dalezios Y. Capogna M. (2012). Neurogliaform cells of amygdala: a source of slow phasic inhibition in the basolateral complex. J. Physiol. 590, 5611–5627. doi: 10.1113/jphysiol.2012.236745, PMID: 22930272 PMC3528981

[ref26] Mate Z. Poles M. Z. Szabo G. Bagyanszki M. Talapka P. Fekete E.. (2013). Spatiotemporal expression pattern of DsRedT3/CCK gene construct during postnatal development of myenteric plexus in transgenic mice. Cell Tissue Res. 352, 199–206. doi: 10.1007/s00441-013-1552-7, PMID: 23370601

[ref27] Matuszewich L. Karney J. J. Carter S. R. Janasik S. P. O'Brien J. L. Friedman R. D. (2007). The delayed effects of chronic unpredictable stress on anxiety measures. Physiol. Behav. 90, 674–681. doi: 10.1016/j.physbeh.2006.12.006, PMID: 17275043 PMC1931411

[ref28] Matyas F. Freund T. F. Gulyas A. I. (2004). Convergence of excitatory and inhibitory inputs onto CCK-containing basket cells in the CA1 area of the rat hippocampus. Eur. J. Neurosci. 19, 1243–1256. doi: 10.1111/j.1460-9568.2004.03225.x, PMID: 15016082

[ref29] Mcdonald A. J. Muller J. F. Mascagni F. (2002). GABAergic innervation of alpha type ii calcium/calmodulin-dependent protein kinase immunoreactive pyramidal neurons in the rat basolateral amygdala. J. Comp. Neurol. 446, 199–218. doi: 10.1002/cne.10204 11932937

[ref30] Meyer A. H. Katona I. Blatow M. Rozov A. Monyer H. (2002). In vivo labeling of parvalbumin-positive interneurons and analysis of electrical coupling in identified neurons. J. Neurosci. 22, 7055–7064. doi: 10.1523/JNEUROSCI.22-16-07055.2002, PMID: 12177202 PMC6757887

[ref31] Miles R. Toth K. Gulyás A. I. Hajos N. Freund T. F. (1996). Differences between somatic and dendritic inhibition in the hippocampus. Neuron 16, 815–823. doi: 10.1016/S0896-6273(00)80101-4 8607999

[ref32] Mineur Y. S. Mose T. N. Maibom K. L. Pittenger S. T. Soares A. R. Wu H.. (2022). Ach signaling modulates activity of the GABAergic signaling network in the basolateral amygdala and behavior in stress-relevant paradigms. Mol. Psychiatry 27, 4918–4927. doi: 10.1038/s41380-022-01749-7, PMID: 36050437 PMC10718266

[ref33] Pape H. C. Pare D. (2010). Plastic synaptic networks of the amygdala for the acquisition, expression, and extinction of conditioned fear. Physiol. Rev. 90, 419–463. doi: 10.1152/physrev.00037.2009, PMID: 20393190 PMC2856122

[ref34] Paxinos G. (2012). Paxinos and Franklin's the mouse brain in stereotaxic coordinates, San Diego, Academic Press.

[ref35] Peterfi Z. Urban G. M. Papp O. I. Nemeth B. Monyer H. Szabo G.. (2012). Endocannabinoid-mediated long-term depression of afferent excitatory synapses in hippocampal pyramidal cells and GABAergic interneurons. J. Neurosci. 32, 14448–14463. doi: 10.1523/JNEUROSCI.1676-12.2012, PMID: 23055515 PMC3494839

[ref36] Pitler T. A. Alger B. E. (1992). Postsynaptic spike firing reduces synaptic GABAA responses in hippocampal pyramidal cells. J. Neurosci. 12, 4122–4132. doi: 10.1523/JNEUROSCI.12-10-04122.1992, PMID: 1403103 PMC6575956

[ref37] Polepalli J. S. Gooch H. Sah P. (2020). Diversity of interneurons in the lateral and basal amygdala. Npj Sci. Learn. 5:10. doi: 10.1038/s41539-020-0071-z, PMID: 32802405 PMC7400739

[ref38] Romanski L. M. Clugnet M. C. Bordi F. Ledoux J. E. (1993). Somatosensory and auditory convergence in the lateral nucleus of the amygdala. Behav. Neurosci. 107, 444–450. doi: 10.1037/0735-7044.107.3.444, PMID: 8329134

[ref39] Rovira-Esteban L. Peterfi Z. Vikor A. Mate Z. Szabo G. Hajos N. (2017). Morphological and physiological properties of CCK/CB1R-expressing interneurons in the basal amygdala. Brain Struct. Funct. 222, 3543–3565. doi: 10.1007/s00429-017-1417-z 28391401

[ref40] Senn V. Wolff S. B. Herry C. Grenier F. Ehrlich I. Grundemann J.. (2014). Long-range connectivity defines behavioral specificity of amygdala neurons. Neuron 81, 428–437. doi: 10.1016/j.neuron.2013.11.006, PMID: 24462103

[ref41] Shultz B. Farkash A. Collins B. Mohammadmirzaei N. Knox D. (2022). Fear learning-induced changes in AMPAR and NMDAR expression in the fear circuit. Learn. Mem. 29, 83–92. doi: 10.1101/lm.053525.121, PMID: 35169047 PMC8852224

[ref42] Somogyi P. (1977). A specific ‘axo-axonal’ interneuron in the visual cortex of the rat. Brain Res. 136, 345–350. doi: 10.1016/0006-8993(77)90808-3 922488

[ref43] Trouche S. Sasaki J. M. Tu T. Reijmers L. G. (2013). Fear extinction causes target-specific remodeling of perisomatic inhibitory synapses. Neuron 80, 1054–1065. doi: 10.1016/j.neuron.2013.07.047, PMID: 24183705 PMC3840076

[ref44] Vereczki V. K. Müller K. Krizsán E. Máté Z. Fekete Z. Rovira-Esteban L.. (2021). Total number and ratio of GABAergic neuron types in the mouse lateral and basal amygdala. bioRxiv 41, 4575–4595. doi: 10.1523/JNEUROSCI.2700-20.2021 PMC8260245 33837051

[ref45] Vereczki V. K. Veres J. M. Muller K. Nagy G. A. Racz B. Barsy B.. (2016). Synaptic organization of perisomatic GABAergic inputs onto the principal cells of the mouse basolateral amygdala. Front. Neuroanat. 10:20. doi: 10.3389/fnana.2016.00020 27013983 PMC4779893

[ref46] Veres J. M. Nagy G. A. Hajos N. (2017). Perisomatic GABAergic synapses of basket cells effectively control principal neuron activity in amygdala networks. eLife 6:e20721. doi: 10.7554/eLife.20721, PMID: 28060701 PMC5218536

[ref47] Veres J. M. Nagy G. A. Vereczki V. K. Andrasi T. Hajos N. (2014). Strategically positioned inhibitory synapses of axo-axonic cells potently control principal neuron spiking in the basolateral amygdala. J. Neurosci. Off. J. Soc. Neurosci. 34, 16194–16206. doi: 10.1523/JNEUROSCI.2232-14.2014, PMID: 25471561 PMC6608493

[ref48] Wilson R. I. Nicoll R. A. (2001). Endogenous cannabinoids mediate retrograde signalling at hippocampal synapses. Nature 410, 588–592. doi: 10.1038/35069076, PMID: 11279497

[ref49] Wolff S. B. Grundemann J. Tovote P. Krabbe S. Jacobson G. A. Muller C.. (2014). Amygdala interneuron subtypes control fear learning through disinhibition. Nature 509, 453–458. doi: 10.1038/nature13258, PMID: 24814341

[ref50] Woodruff A. R. Sah P. (2007). Inhibition and synchronization of basal amygdala principal neuron spiking by parvalbumin-positive interneurons. J. Neurophysiol. 98, 2956–2961. doi: 10.1152/jn.00739.2007, PMID: 17715201

[ref51] Zhu P. J. Lovinger D. M. (2005). Retrograde endocannabinoid signaling in a postsynaptic neuron/synaptic bouton preparation from basolateral amygdala. J. Neurosci. 25, 6199–6207. doi: 10.1523/JNEUROSCI.1148-05.2005, PMID: 15987949 PMC1352167

